# Advancing C-V2X for Level 5 Autonomous Driving from the Perspective of 3GPP Standards

**DOI:** 10.3390/s23042261

**Published:** 2023-02-17

**Authors:** Muhammad Jalal Khan, Manzoor Ahmed Khan, Sumbal Malik, Parag Kulkarni, Najla Alkaabi, Obaid Ullah, Hesham El-Sayed, Amir Ahmed, Sherzod Turaev

**Affiliations:** 1 College of Information Technology, United Arab Emirates University, Abu Dhabi 15551, United Arab Emirates; 2 Emirates Center for Mobility Research (ECMR), United Arab Emirates University, Abu Dhabi 15551, United Arab Emirates

**Keywords:** 4G, 5G, V2X, 3GPP, standards

## Abstract

Cellular vehicle-to-everything (C-V2X) is one of the enabling vehicular communication technologies gaining momentum from the standardization bodies, industry, and researchers aiming to realize fully autonomous driving and intelligent transportation systems. The 3rd Generation Partnership Project (3GPP) standardization body has actively been developing the standards evolving from 4G-V2X to 5G-V2X providing ultra-reliable low-latency communications and higher throughput to deliver the solutions for advanced C-V2X services. In this survey, we analyze the 3GPP standard documents relevant to V2X communication to present the complete vision of 3GPP-enabled C-V2X. To better equip the readers with knowledge of the topic, we describe the underlying concepts and an overview of the evolution of 3GPP C-V2X standardization. Furthermore, we provide the details of the enabling concepts for V2X support by 3GPP. In this connection, we carry out an exhaustive study of the 3GPP standard documents and provide a logical taxonomy of C-V2X related 3GPP standard documents divided into three categories: 4G, 4G & 5G, and 5G based V2X services. We provide a detailed analysis of these categories discussing the system architecture, network support, key issues, and potential solution approaches supported by the 3GPP. We also highlight the gap and the need for intelligence in the execution of different operations to enable the use-case scenarios of Level-5 autonomous driving. We believe, the paper will equip readers to comprehend the technological standards for the delivery of different ITS services of the higher level of autonomous driving.

## 1. Introduction

Autonomous driving is no longer hype but a reality. Industry, the research community, and other stakeholders including authorities have been working on realizing the objectives of different levels of autonomous driving (AD). This is evident by the plethora of research contributions and activities around the globe. Standardization bodies have also been active in pushing the core research and vision of the stakeholders toward such realizations. Amongst others the 3rd Generation Partnership Project (3GPP) has been contributing to the technological standardization for realizing different AD use cases. The focus of standardization activities remained across the different communication-relevant solution components of AD. This was obviously boosted after the major original equipment manufacturers (OEMs) and regions such as the USA and European Union decided on 5th-generation (5G) as the technology of choice for cellular vehicle-to-everything (C-V2X). This also shifted the paradigm from simple V2X communication to a number of communication modes including Vehicle-to-Vehicle (V2V), Vehicle-to-Pedestrian (V2P), Vehicle-to-Network (V2N), Vehicle-to-Infrastructure (V2I), Vehicle-to-Network-to-Pedestrian (V2N2P), Vehicle-to-Infrastructure-to-Network (V2I2N), etc. Obviously, the C-V2X is a concept known to the community and industry prior to the design and commercialization of 5G, which was then known as long-term-evolution-V2X (LTE-V2X). Meaning thereby, vehicle-to-everything (V2X) communication supported by 4th-generation (4G) mobile network technology. Standardization and commercialization of 5G mobile network technologies have pretty much addressed the most crucial issues of vehicular communication (specific to AD) enabling the stakeholders to realize the design goals ofLevel-5 (L-5) AD.

C-V2X has attracted huge attention in the recent past from industry, academia, and research community, the consequence of which is a large number of standard documents, design approaches, technical specifications, and reports on key issues and solution components. Obviously, such resources are not mutually exclusive and mostly overlap in contents (though the degree of overlap may vary). The current literature does not provide a simple yet easy-to-understand categorization of the technical specifications and reports. For example, Harounabadi et al. [1] provide general aspects rather than a full overview to cover all aspects of V2X applications. One may see the obvious challenge here i.e., a problem similar to finding a needle in a haystack. Finding the key support areas for V2X services is yet another open challenge to be resolved. To address these and similar challenges, we extensively analyze the 3GPP standards relevant to AD in general and C-V2X communication in particular. It is important to reiterate that 3GPP is a standardization body and a consortium of seven telecommunication organizations that develop standards. Therefore, we aim to analyze 3GPP’s reports and specifications for advanced cellular communication technologies i.e., 4G and 5G. With the help of these technologies, 3GPP provides various concepts that help to realize the true potential of vehicular communication for higher levels of autonomous driving. Furthermore, we performed categorization of the technical documents, which is also inspired by the flow of 3GPP file server documentation, topics and scope of each document, and major communication platforms i.e., 4G and 5G mentioned in each technical document.

### Case of Interest of This Paper

The 3GPP has been supporting vehicular communication through 4G and 5G technologies. In this paper, we focus on analyzing and simplifying the 3GPP contributions in relation to the support provided for realizing the higher level of C-V2X applications. We present our case of interest as follows:

- 3GPP standardizes for a large set of use-case scenarios when it comes to supporting vehicular communication (in general) and C-V2X in particular. One major contribution of this work is to conduct extensive analyses of the 3GPP C-V2X standards, which are compiled in a way to guide better the readers, research community, and industry.
- The 3GPP standard documents provide various key issues and their potential solutions, which will help in realizing the true potential of a higher level of C-V2X communication. A literature survey shows that there is a clear lack of resources that present most (if not all) key issues, assumptions, and solutions. Hence, in this article, we elaborate on all of these and present easy-to-comprehend content by discussing these together and their roles in achieving the L-5 AD. We believe this will equip the readers and researchers in a way allowing them to study the right potential solutions for the challenges of their interest. This will assist in evolving the research work leading towards achieving the solutions of higher autonomy levels.
- 3GPP is a large body of various strong partners and there exists a large amount of support for different categories of V2X communication. Therefore, we performed an exhaustive study of 3GPP standard documents and presented a categorization of the documentation about V2X services.
- The available standard documents tend to introduce ignorance of the readers. Therefore, we provide an easy-to-follow discussion for the readers about use cases, challenges, communication requirements, and potential solutions. This simplified version of the discussion aims to build readers’ involvement by making their interest in 3GPP standardization.
- The work provided by 3GPP for standardized V2X communication is complex to understand and therefore, we provide a simplified structure of 3GPP documentation and technological standards of intelligent transportation systems (ITS) services to achieve L-5 AD.
- The current standpoint of 3GPP standardization for V2X services places little or no emphasis on the intelligence part in vehicular communication. In this regard, we highlighted the missing gaps for introducing intelligence in the execution of different network operations for enabling the use-case scenarios.

The rest of the paper is structured as follows. Section 2 presents the background of C-V2X and 3GPP, followed by the taxonomy of C-V2X related 3GPP standard documents in Section 3. The support for 4G & 5G, and 5G based V2X services are presented in the Section 4, and Section 5, respectively. Section 6 presents the network data analytics function, followed by the future directions in Section 7. Finally, Section 8 elucidates the conclusion of the paper.

## 2. Background

In this part, we equip the readers with the necessary background information to comprehend the contents of this research work. We discuss the role of C-V2X in AD, modes of C-V2X communication, and an overview of 3GPP releases.

### 2.1. Cellular-V2X for Autonomous Driving

Communication plays a crucial role in attaining the objectives of AD, which is why the stakeholders assume that the current “connected and automated vehicles” (CAVs) will evolve into autonomous vehicles (AVs). Needless to mention for the vehicles to autonomously operate in any environment, they should be able to communicate with other vehicles and objects. Initially, such communication was based on WiFi-based technology (i.e., IEEE 802.11p/Dedicated Short Range Communication (DSRC)) [2]. However, the concept of the cellular-V2X (C-V2X) is relatively new, where the V2X communication was realized through cellular technology i.e., LTE / 4G. C-V2X is contributed by 3GPP as an alternative to DSRC, which operates in the 5.9 GHz frequency band, a band allocated around the globe for device-to-device/short-range communication. For instance, the authors of [3] describe a complex simulation study that compares both DSRC and LTE (mode 1 and mode 3) technology in a simulated urban environment.

The cellular technologies of 3GPP support the communications between vehicles such that vehicles send and receive signals either through using 4G (LTE) and/or 5G. In addition, this communication process also includes connectivity with network and infrastructure either with fixed or dynamic objects. The C-V2X technology of 3GPP uses a common frequency band reserved for ITS applications and supports the realization of V2X requirements. Through its various releases, as presented and discussed throughout this paper, 3GPP showcased the support in realizing C-V2X through 4G (LTE), 5G, and beyond 5G (B5G) communication technologies. The efforts for the C-V2X standard introduced by 3GPP were significant steps towards achieving the design goals of AD and other automotive verticals. The support for the automotive industry was made possible through enhancing vehicular communication by expanding 3GPP’s 4G (LTE) platform and later by Non-Standalone (NSA) 5G technology. Hence, 3GPP Release 14 (*Rel-14*) started presenting operational scenarios for C-V2X standard.

The C-V2X technology performs well in high-dense traffic areas and provides high reliability and real-timeliness in communication between entities i.e., vehicles, pedestrians, networks, and infrastructures. Focusing on Standalone (SA) 5G and future communications, the C-V2X helps and supports both ranges of transmissions i.e., short- and long-range communications. C-V2X is expected to be instrumental in enabling more sophisticated ITS and infotainment services when it comes to autonomous vehicles paradigm such as vehicle diagnostics, connected infotainment, pay-as-you-drive insurance, autonomous driving, collaborative autonomous driving [4], platooning [5,6,7], remote driving, and other safety features, etc., by leveraging the mix-range module [8]. However, the true potential of C-V2X technology may not be explored unless the proper coordination amongst the right stakeholders is achieved. These entities and their interaction generate a large amount of data and distributed decision engines, which require reliable and fitting communication bit pipes. C-V2X and 5G mobile network is expected to pave the path towards achieving the objectives of L-5 AD. For instance, to achieve enhanced perception and informed decision-making, 5G pledges the enhanced mobile broadband and ultra-reliable low-latency communication (URLLC) pillars.

### 2.2. Modes of C-V2X Communications

C-V2X manifests in two forms: (1) direct communication between devices referred to as device-to-device mode; (2) indirect communication between devices through the underlying network referred to as device-to-network mode. These two different modes introduce V2V, V2P, V2I, and V2N communication types. The following provides a brief description of the two modes of C-V2X technology.

- **Device-to-Device Mode:** This mode of C-V2X communication deals with enabling the communication bit-pipes between vehicles (V2V), pedestrians (V2P), and infrastructures (V2I). Hence, the vehicular communication types i.e., V2V, V2P, and V2I are realized by device-to-device mode furthering the direct communication between devices (vehicles, pedestrians, and infrastructures are considered as devices) [9].
- **Device-to-Network Mode:** This mode of C-V2X communication enables the communication of bit-pipes between devices and network elements/entities. V2N is realized by this mode of C-V2X implementation. Hence, the end-user (driver) is able to achieve the advantages of network and cloud services. The V2N plays an essential role in completing the picture of an end-to-end solution for various verticals based on C-V2X communications.

In view of the above communication modes and considering all devices will be in IoT networks in the future, we would like to mention the essential difference between V2N and V2X networks. Since V2X is a universal set of the vehicle-to-everything network, it includes V2V, V2I, V2N, V2P, etc. Meaning thereby, V2X is a broader term capturing vehicle communication with anything, whereas V2N is a narrow term capturing the communication between vehicles and the network. In this connection, the IoT networks would mean the network that connects vehicles. If we consider the vehicle as one of the things, the network will connect the vehicle with other vehicles and/or things. In this paper, we keep our focus open where vehicles can communicate with anything (i.e., V2X) through different modes of communication.

### 2.3. An Overview of 3GPP Releases

The ongoing efforts for smarter management of traffic and use of transport networks require ITS-enabled innovative services. Amongst others, these services assist road users (vehicles, pedestrians, cyclists, etc.) in making informed decisions about safety and mobility. For instance, traffic intensity in road segments may be controlled by dynamically adapting the traffic light control timers and adjusting the speed limits, enabling an AV to proactively plan a maneuver to avoid a collision with an object(s) on the road, etc. Although work in ITS has been focusing on standardizing the communication interfaces, security and privacy are some of the main issues associated with the development and deployment of ITS applications. To support the ITS applications, 3GPP started including major elements for vehicular communications i.e., “Internet on Wheels” [10]. Starting from *Rel-14* [11], the communication technologies of 3GPP i.e., 4G (before) and 5G (now) have had a great influence on the success of C-V2X applications.

The 3GPP contains technical specification groups (TSG) i.e., core network & terminals (CT), service & system aspects (SA), and radio access network (RAN). These TSGs provide specifications and technologies as a result of technical reports, which are released from time to time. In release 14, 3GPP started supporting V2X services and the current releases now discuss the intelligence for V2X use-case scenarios. Following the 3GPP standardized technologies and their selection can be challenging due to the sheer number of technical documents and the difficulty of navigating through each one of them. Hence, it can become a daunting task. Therefore, we reduce the complexity by finding the relevant standard documents for V2X services. The current releases of 3GPP standards are realizing the successful transition from 802.11p or DSRC to 4G-V2X communication. *Rel-14* was one of the early releases, which focused on vehicular communication. Although the need for 5G was initially discussed in *Rel-14*, 5G Phase-1 of 5G system (5GS) started in *Rel-15* [12]. *Rel-14* supports V2X communication through enhancing the 4G technology by evolved packet system (EPS) [13]. Since the V2X gained support from EPS in *Rel-14*, some technical specifications from *Rel-15* introduced enhancements to V2X (eV2X) considering the future of V2X with 5G New-Radio (NR). Furthermore, advanced driving, remote driving, extended sensors, and vehicle platooning are supported in *Rel-14* and *Rel-15*.

The updated version of the 5G system (5GS: 5G Phase 2) presented in *Rel-16* (*TR 21.916*) extends the 5G specifications i.e., architectural aspects and service-oriented functionalities for V2X. Hence, *Rel-16*’s 5G supports the advanced V2X services and the authors from [13] analyzed the architectural status of *Rel-16*. The *Rel-16* (current) [14] and *Rel-17* (future) [15] releases discuss NR sidelink communication and its improvements [1], which targets important aspects i.e., resource allocation, power efficiency, high QoS, etc., for successful V2X communication. In addition, new use-case scenarios are under study in *Rel-17* and *Rel-18* [16] e.g., AI-based sidelink communication for devices, communication between robots and machines.

**Figure 1:**
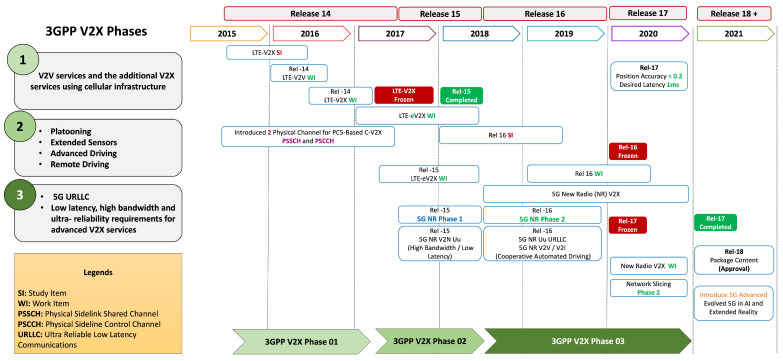
Evolution of 3GPP C-V2X Standardization.

The C-V2X evolution is presented in Figure 1. The V2V standards and basic safety services are delivered in *Rel-14* phase 1. To provide support for eV2X services complementary to *Rel-14*, the *Rel-15* phase 2 built a stable LTE-V2X (4G-V2X) ecosystem by providing services i.e., platooning, extended sensors, advanced driving, and remote driving. In addition, the *Rel-15* introduced various new technical features such as longer range, higher-order modulation (64QAM), lower latency (10ms min), higher reliability, and carrier aggregation up to eight component carriers. Advancement to 5G NR-V2X (5G-V2X), phase 3 is designed to deliver support for the advanced V2X (aV2X) services with lower latency, ultra-reliability, and higher throughput. The 5G-NR phase 1 started in *Rel-15* and the 5G-NR phase 2 in *Rel-16*. The 5G URLLC network slicing supports the advanced functions of Level-3 and Level-4 AD with a higher quality-of-service (QoS) profile. Though the 3GPP NR *Rel-17* provides new sidelink communication relaying architecture aiming to support some of the aV2X services, the *Rel-18*, yet to be published, aims to introduce new features and services such as 5G advanced, evolved 5G in AI and extended reality, etc. The depicted V2X phases in the evolution provide a clear path to the research community for putting efforts in the right direction toward providing solution components for V2X services.

## 3. Taxonomy of C-V2X Related 3GPP Standard Documents

**Figure 2:**
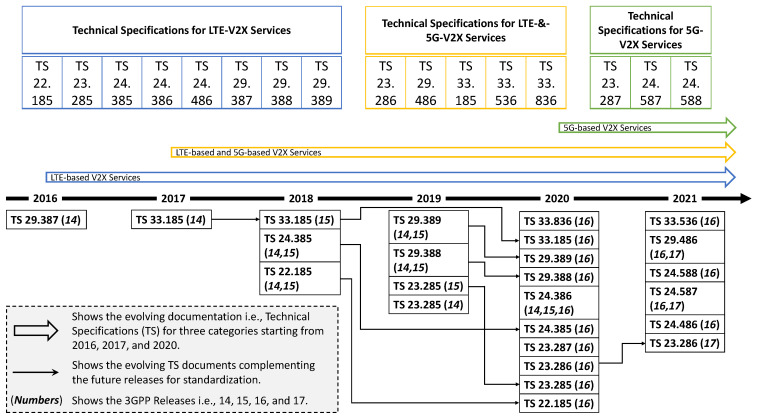
Evolution of 3GPP’s Technical Specifications.

**Figure 3:**
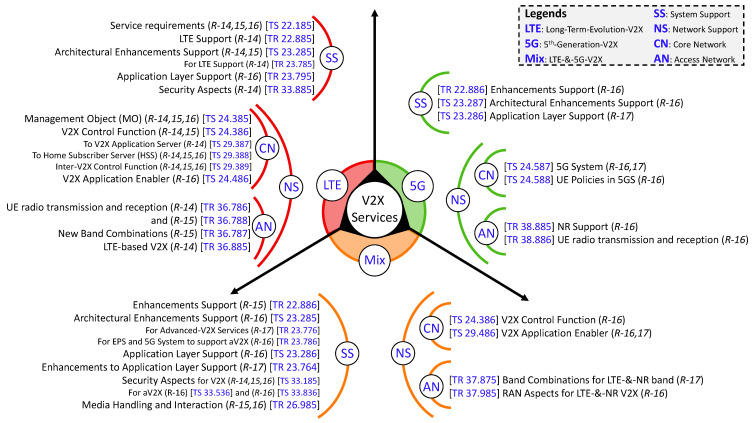
System and Network Support for V2X Services.

With the view to align the standard documents with current trends and guide the readers to the right documents, we suggest a simple but descriptive taxonomy of the standard documents in this section. In this regard, we present Table 1, Figure 2 and Figure 3. The idea is to:

- Categorize the standard documents following the natural evolution path of communication technologies,
- Evolution of technical specifications showcasing the dependency among specification documents,
- Decompose the services on the system and network segments including core and access network,
- Map the standard documents to the aforementioned system and network segments.

We believe that the contributed categorization and taxonomy will significantly aid the stakeholders in their pursuits. The standard documents encamp rich V2X relevant guidelines for future scenarios, however, distributed across a wide range of documents. As can be seen in Table 1, we ensure the arrangement of relevant documents in terms of their technological evolution e.g., 4G, 4G & 5G, and 5G based V2X services. Furthermore, we have also arranged the documents according to the release number under each technological category, which clearly visualizes the continuation and focus of various topics in different releases. The use-cases and services (from *TR 22.885*) support V2X communication types and address the safety and non-safety issues through 4G technology [17]. Therefore, it goes without saying that the technical report (*TR 22.885*) is the foundational building-block for identifying 4G V2X use-cases and their potential requirements. Since each 4G V2X use-case provides description, pre-conditions, service flows, post-conditions, and potential requirements, the readers are encouraged to go through *TR 22.885* as we simply list few use-cases here owing to the page limitation i.e., emergency vehicle warning, road safety services, wrong-way driving warning, curve speed warning, etc. Furthermore, the report also presented coverage, spectrum, security, mobility, future-proofness, and deployment considerations and listed various important V2X deployment example scenarios. Hence, the identified use cases and other important details complement the 3GPP standardization documents for 4G (before) and 5G (now) V2X communication services.

To equip the readers with a simplified version of the technical specification (TS) documents evolution for C-V2X services starting from 2016 until 2021, Figure 2 presents technical specifications’ categorization fed from Table 1 and yearly evolution of the produced specifications. The annual division of the TSs highlights the updated production of documents. For example, the majority of the updated documents are produced in the year 2020, which are complemented by documents from the years 2018 and 2019. It can be noted that *Rel-16* supported the realization of V2X services by producing almost half of the TS documents, which are complemented by TS documents from *Rel-14* and *Rel-15*. However, the *Rel-16*’s TS documents outreached the support for 5G V2X services. Therefore, *Rel-17*’s TS documents (complemented by *Rel-16*) started addressing the support for V2X service through 5G technology.

The 3GPP includes three important technical specification groups (TSG) i.e., radio access network (RAN) [18], service & system aspects (SA), and core network & terminals (CT) [19]. These TSGs focus on requirements, functions, interfaces, architectures, execution environments, etc., for supporting V2X services. Inspired by the TSGs and missing support of literature, we decompose the C-V2X-based 3GPP technical documents into system and network (including core and access network) support. Figure 3 shows a more precise yet easy-to-understand system and network support categories for cellular technologies over three axes, where each axis presents different services based on different cellular technologies i.e., 4G, 4G & 5G (Mix), and 5G. The design also dictates the fact that there exists dependency among technical documents.

The current version of the design is presenting the V2X services in three coordinates, and the future version of this research work may consider the exact positioning of technical documents over axes with some functions making a holistic approach for support categorization. So, another visualization can be provided by pointing out how much the documents contribute to other parts. Hence, we focused on creative and effective-to-understand support categorization design. The system support (SS) includes service requirements, architectural enhancements, application layer, security aspects, media handling & interaction, and other enhancements support for 4G and 5G V2X services. A total of 19 technical specifications and reports are produced for system support. Similarly, the core network (CN) support includes a control function, application server, application enabler, 5G system, UE policies, etc., for C-V2X services, and 10 technical documents are produced for CN support. Last but not least, the access network (AN) support includes band combination, RAN aspects, UE radio transmission & reception, etc., for 4G and 5G V2X services. A total of 8 technical documents are produced for AN support. The support categorization provides an easy-to-navigate approach for readers to equip themselves with an exact support category rather than following the puzzling structure of standardization documentation.

## 4. 4G and NSA-Based 5G V2X Services

The evolution of V2X from 4G (LTE) to 5G includes an important period where both technologies have been playing a hybrid role aiming to achieve support for V2X services. In terms of architecture and technology, NSA-based 5G technology is backward-compatible with 4G technology. Therefore, we refer to NSA-based 5G as 5G in this section. In what follows next, we focus on analyzing the system and network support for 3GPP’s 4G and NSA-based 5G V2X services.

### 4.1. System Support

The V2X services continue to receive 4G and 5G-based system support from 3GPP standardization. In this regard, various enhancements are produced for enhancing V2X (eV2X) and advancing V2X (aV2X) services support. These enhancements have been addressing architectures, application layers, security aspects, media handling, etc.

#### 4.1.1. Enhancements Support

The 3GPP discusses the enhancements to support V2X services aiming to meet the increasing services requirements of V2X applications. The enhancements support discussed in the document (*TR 22.886 Rel-15*) provides various use-cases for eV2X services such as cooperative perception, lane change, information sharing, cooperative driving, remote driving, platooning, video-sharing, and composition for enhanced V2X scenarios. However, the majority of use cases are backed/sustained by the identification of basic use cases (from *TR 22.885*) and their potential service requirements. Hence, the old and newly identified use cases make use of 4G and 5G NR communication technologies to meet the communication and other supports for eV2X services. Moreover, the specified potential requirements are used for communicating warning messages to the neighboring vehicles through EPS enhancements confirming the use-cases evolution as a necessary step for emerging eV2X applications. To help the readers with an easy understanding through an abstract view, the 3GPP reported a mapping between use-cases to form use-case groups. These use case groups further help in the system support for 5G V2X services discussed in Section 5.

#### 4.1.2. Architectural Enhancements Support

To facilitate vehicular communications (via PC5 and Uu) for eV2X services, the specification document (*TS 23.285 Rel-16*) provides the architecture reference model enhancements supporting the service requirements and use cases stated in earlier specification documents (i.e., *TS 22.185* and *TS 22.186*). The older releases of the specification (*TS 23.285 Rel-14* and *Rel-15*) did not focus on the enhancements for 5G such as EPC controlled NR PC5 operations, support of NR PC5 quality-of-service (QoS) under EPC, NR PC5 QoS modeling, etc. These enhancements introduced advancements for V2X applications, known as advanced-V2X (aV2X) services. The aV2X services are comprised of advanced scenarios such as advanced driving, remote driving, platooning, extended sensors, general aspects, and vehicle QoS support. Therefore, to summarize the support for each communication scenario, we present the performance requirements provided by 3GPP in the *(TS 22.186)* in Table 2. In what follows next, we provide an overview of the support for aV2X services available in *Rel-16* and *Rel-17*.

3GPP discusses the architectural enhancements for EPS and 5G systems in (*TR 23.786 Rel-16*) to support V2X services. Moreover, the TR also specifies the assumptions, requirements, and 15 key issues. Some of the important key issues are network slicing, interworking for eV2X, edge computing, NR-based PC5 communication, sensor sharing over PC5, service authorization, provisioning, etc. Furthermore, the TR provides 29 solution approaches and some of the potential solutions are network slicing, application function influence-based edge computing for V2X, interworking for eV2X, eV2X impacts to 5GC procedures, and network-controlled QoS mechanism for PC5 communication. Four variants of eV2X architecture including architecture reference models have also been discussed.

In *Rel-17*, the architectural enhancements i.e., assumptions and requirements for advancing V2X services are provided in the technical report (*TR 23.776 Rel-17*). In this regard, the 5GS enhancements are investigated for advanced operations and applications of eV2X services. For example, power-efficient communication between UEs and pedestrians. The key issues identified in *Rel-16*’s technical report (*TR 23.786*) are considered with new key issues in *Rel-17*’s technical report (*TR 23.776*). One of the important issues is the support of QoS-aware NR PC5 power efficiency for pedestrian UEs. Similarly, some of the potential solutions are QoS-aware power-efficient PC5 communication for pedestrian UEs, V2P parameter provisioning, QoS support for V2X messages sent by pedestrian UEs, etc., reported in the technical document (*TR 23.776 Rel-17*). It goes without saying that 3GPP standardization will continue supporting V2X services with architectural enhancements in future releases i.e., *Rel-18*.

#### 4.1.3. Application Layer Support

The 3GPP (*TS 23.286 Rel-16*) provides functional architecture, procedures, and information flows enabling the application layer of 4G & 5G based V2X applications to make efficient utilization and deployment of V2X services. Meaning thereby, the V2X VAE helps the application layer in deploying the V2X applications. However, this support depends on the fulfillment of the architectural requirements for group communication, dynamic groups, file distribution capability, message distribution, and service continuity as outlined in the aforesaid specification. Therefore, a VAE functional model and its entities are described to address the support for 4G & 5G V2X services. These entities communicate with each other through various internal interfaces such as V1, V5, Vs, Vc, PC5, Uu, etc., and external reference points i.e., V2, Rx, T8, etc., (the descriptions for these interfaces can be found in (*TS 23.286 Rel-16*)). For the deployment of the VAE functional model, one may choose between centralized and distributed deployment of VAE servers in the V2X service provider domain. The application layer needs to identify the user, UE, service, group, etc., in V2X communication. In this regard, the stated specification provides identities for the stated actors. For information flows, the entities and actors perform various operations. We list some of the important procedures i.e., UE registration, location tracking, message delivery, file distribution, network monitoring, switching modes of operations, service delivery, resource management, QoS monitoring, etc. To avail the full use of these operations, the specification (*TS 23.286 Rel-16*) provides details over VAE layer Application Programming Interfaces (APIs) focusing on server APIs.

The V2X application layer architecture, discussed above, assisted in addressing the utilization and deployment of services through the VAE functional model. Since these services were produced in *Rel-16* of the specification (*TS 23.286*), there exists a need for enhancing the architecture for future releases. In this regard, the technical document (*TR 23.764*) in *Rel-17* reported and identified the enhancements to application layer support for V2X services. Moreover, the report includes key issues (16), solutions (18), architectural requirements, and an analysis of V2X standards. Some of the important key issues are eV2X application QoS requirements for V2X communication over PC5 and Uu, V2X control and message distribution over Uu, UE-to-UE application layer relay, and V2X UE measurement reporting to the VAE server. Whereas some key issues only highlight the demand for various supports, i.e., network slicing, teleoperated driving, communication modes, switching modes for communication types, service providers, and dynamic information for high-definition (HD) maps. Some of the important requirements i.e., reliability, data rate, transmission rate, maximum latency, communication range, etc., can be found in the detailed descriptions of these key issues.

Apart from the requirements listed for key issues, the 3GPP reported architectural requirements for V2X message distribution, eV2X QoS monitoring & control by the application layer, and coordination based on EPS and 5GS connectivity in (*TR 23.764 Rel-16*) for addressing the key issues. In addition, the important solutions for key issues are NR-PC5 QoS monitoring and control, monitoring and control of QoS for eV2X communications, UE-to-UE broadcast configuration and delivery by VAE layer, UE-initiated session-oriented service establishment support, communication configuration for switching modes, etc. The discussed enhancements report also provides an analysis of the impactful functions of the V2X application layer and declares two internal reference points (interfaces), i.e., V1 and V5 as out of scope of technical specification (*TS 23.287 Rel-16*), which is discussed in Section 5. It goes without saying that these key issues and their corresponding solutions hold importance in addressing future challenges related to V2X services. Hopefully, the research community working with V2X services and applications will witness huge support in enhancing and advancing the V2X application layer architecture in the announced future release i.e., *Rel-18*.

#### 4.1.4. Security Aspects Support

The 3GPP support for 4G & 5G V2X services with regard to enhancements in either V2X architectures or V2X application layer confirms huge improvements for efficient and effective utilization of cellular technologies and vehicular communications as documented in various technical reports and specifications. Security is yet another important aspect and is required for secure vehicular communication between vehicles, pedestrians, networks, and other related entities. In this regard, the technical specification (*TS 33.185*) of *Rel-14-15-&-16* started supporting security aspects by introducing requirements on the network entities, architectures, procedures, and solutions for supporting secure V2X and aV2X services. In what follows next, we provide an interesting and critical analysis of the security aspects of C-V2X features.

The overall architectural enhancements (specified in (*TS 23.285 Rel-16*)) for vehicular communication services addressing safety and non-safety aspects in 4G & 5G V2X services are a base for introducing security aspects for V2X architectural interfaces. Hence, the research community should note important V2X security requirements for interfaces between network elements, interfaces between UE and V2X control function (V3), an interface between external provider and 3GPP network (MB2), V2X application data, V2X entities secure environment, and privacy. These V2X security requirements will allow the researchers to explore and identify new security paradigms and aspects of V2X features in addition to the current available V2X security solutions. For instance, Hakeem et al. [20] provides a protocol that combines short-size signatures and bilinear pairing cryptography to ensure authentication and privacy. Furthermore, the potential areas where security solutions have been proposed for V2X are: V2X communication between network elements, V2X communication between UE and V2X control function (V3), an interface between the V2X application server and 3GPP network (MB2), and privacy in V2X services. The stated solutions include general descriptions and security procedures (in (*TS 33.185 Rel-16*)) along with other technical specifications that help in achieving the goals of security aspects in V2X services.

The discussed technical specification for security aspects of V2X services encourages the researchers working with 3GPP to introduce other technical specifications (*TS 33.836 Rel-16* and *TS 33.536 Rel-16*), which specify security aspects for eV2X and aV2X services. The former specification document provides details on eV2X system architecture analysis, potential requirements, and solutions evaluations. The considered security aspects i.e., security and privacy for new interfaces in 5G eV2X system architecture, eV2X unicast over PC5, and eV2X group communication over PC5, and their complete descriptions are available in the former specification document. The latter specification document provides requirements to achieve the security for V2X services, procedures to fulfill the specified requirements, and details on 5GS in order to facilitate vehicular communication for V2X services and V2X architecture (available in *TS 23.287*). In addition, it considers security aspects for cases other than those available in the document (*TS 33.185 Rel-16*) i.e., PC5 reference point security. The other important security cases are V2X over reference points i.e., Uu and NR-based PC5. These interfaces are well known in comparison to all other reference points due to their importance in vehicular communication for V2X services. It is also noteworthy, that these interfaces and other reference points are used in the V2X architectures as discussed in document (*TS 23.287*).

The communication modes i.e., unicast and groupcast, over NR PC5 are considered the potential for security impacts in technical specification (*TS 33.836 Rel-16*) because the security for broadcast mode may receive several changes in the upcoming releases. Furthermore, the specification lists many key issues and their potential solutions. Some of the important key issues are security for eV2X unicast and broadcast messages over PC5, privacy protection for unicast, broadcast, and groupcast messages over PC5, security for setting up groupcast, security of the UE service authorization and revocation, etc. Apart from the solutions provided for the above-mentioned key issues, some other important solutions are against V2X UE tracking based on PC5 identifiers, solution for the activation of user plane security in NR PC5 unicast, PC5 layer key derivation using the 5G network keys, solution on minimizing the impact of privacy protection mechanism in the application layer communication, etc. Each of the solutions mentioned here and from the technical specification (*TS 33.836 Rel-16*) contains complete solution details and evaluations. Obviously, when the security aspects for aV2X services based on 4G & 5G technologies will purely follow the 5G architectures, a number of issues are expected.

#### 4.1.5. Media Handling and Interaction Support

For 4G V2X services, the 3GPP supported and focused on simple message delivery for collision avoidance. Apart from many important 4G & 5G V2X use cases, media handling i.e., video sharing and composition scenarios (described in (*TR 22.886 Rel-15*)) is one of the crucial use cases for advanced driving of eV2X services. This dictates the importance of 5G technology fusion with 4G technology to realize and address important and critical V2X use case scenarios. There is no doubt that the architectural and other enhancements (discussed above) are effective in addressing the potential requirements for aV2X services and applications. In the near future, other essential and effective advancements will be produced for future V2X (5G V2X) services. Hence, the research community is encouraged to start focusing on 5G V2X services and use case scenarios.

At the current stage, the support of 4G & 5G communication technologies for V2X services considers advanced use cases and functionalities such as media transmission, video sharing, and visual information utilization. The media handling and interaction reported in the document (*TR 26.985*) provides details (less in *Rel-15* and more in *Rel-16*) for advanced media transmission capabilities using networked visual information. In addition, the *Rel-16* document reports the investigation of procedures for capture, compression, and transmission. For example, the use of a camera and LiDAR for capturing high-resolution perception data and transmitting the compressed version to other UEs. Some of the other important media-based V2X use cases are support for remote driving, information sharing for high/full automated driving, video data sharing for assisted and improved automated driving, teleoperated support, and video composition. Though it is a matter of choice between Uu and PC5 interfaces when realizing media-based V2X use cases, the Uu interface favors QoS and high-bitrate media transmissions. Interestingly, the technical document (*TR 26.985 Rel-16*) describes how to leverage the 5G QoS framework for supporting different traffic and service flows e.g., media streams, maneuver instructions, conversational service, uplink video, and audio flows when realizing remote driving use case. However, the media format i.e., resolution, framerate, bitrate/quality, etc., and adaptation is an open challenge in handling media and interactions. Given the huge importance of media in vehicular operations and services, this could be a useful avenue for researchers to contribute to the development of aV2X services.

### 4.2. Network Support

The 3GPP provides 4G & NSA-based 5G communication technologies-based network support to realize V2X services. The network support is two-fold: core network (CN), which deals with the core network and terminals (CT) support; and access network (AN), which deals with radio access network (RAN) support. In what follows next, we provide an important to-the-point overview of the core and access network support.

#### 4.2.1. Core Network

The core network reflects the technical reports and specifications produced by the core network and terminals (CT-TSG). The CT TSG in 3GPP standardization provides V2X control function and VAE support to 4G & 5G V2X services. In what follows next, we discuss core network support.

##### V2X Control Function Support

The core part of the V2X communication network requires many important functions and protocols. The V2X control function is one of the key functions supported by 4G & 5G V2X communication technologies of 3GPP standardization. In this regard, the 3GPP specification (*TS 24.386 Rel-16*) provides details for a V2X control function, protocols, and other procedures enabling the V2X authorization and communication for 4G & 5G V2X based V2X applications. From an abstract view, the V2X control function supports secure communication, provides configuration parameters, helps with authorization procedures, and keeps an IP address for UEs to be discovered for communication. Similarly, the protocols are used by various interfaces i.e., 4G-Uu, 4G and 5G PC5, V3, etc., of the enhanced architectures discussed in the specification document (*TS 23.285 Rel-16*). This dictates the effective and efficient: V2X authorization, which is between UE and V2X control function; and V2X communication, which is between UEs, and/or between UE and V2X application server. The other procedures also help control the function for transmission and reception of V2X communication messages over the interfaces discussed above.

##### V2X Application Enabler (VAE) Support

The main role of VAE is to support the efficient utilization and deployment of V2X applications and services. Apart from the network-associated discussions, we provided an overview of VAE and various architectural interfaces for realizing V2X services from the 3GPP technical specification (*TS 23.286 Rel-16*) in the application layer under system support. However, the 3GPP also provides core network supports through various VAE services i.e., message delivery, file distribution, application requirement (e.g., reserve and notify network resource), dynamic group, service continuity, etc., and their operations in the technical specification (*TS 29.486*) of *Rel-16* and *Rel-17*. In addition, the defined protocol and data model helps in: VAE server and V2X application server communication through Vs interface; and VAE servers communication through the VAE-E interface. The stated VAE services include APIs for the V2X applications to communicate with the VAE server. The APIs can be used by the researchers to make good use of VAE servers and V2X applications. Apart from this usage, the researchers are encouraged to propose new APIs for VAE network-related services.

#### 4.2.2. Access Network

The access network describes the 3GPP standardization reports and specifications of the radio access network (RAN-TSG). Apart from other supports, band combinations and RAN aspects for 4G & 5G band and V2X are supported by the access network.

##### Band Combinations Support for 4G & 5G Band

The frequency bands of the access network for 4G V2X service from the technical report (*TR 36.787 Rel-15*) play a vital role in completing V2X operations. Other band combinations are required for not only concurrent operations over 4G interfaces but concurrent operations over 4G & 5G interfaces i.e., 5G/4G Uu bands/band combinations and 5G/4G PC5 band as reported in the technical document (*TR 37.875 Rel-17*). Apart from other extensions reported in the technical document, the following three scenarios for concurrent operations can be considered when a single band is selected i.e., 4G-Uu band and 5G-PC5 band; 5G-Uu band and 5G-PC5 band; and 5G-Uu and 4G-PC5 band. Other band combinations and the given scenarios provide enhancements for fulfilling the growth of the 4G & 5G V2X ecosystem. In this connection, the available RAN aspects from technical report (*TR 37.985 Rel-16*) are important for expanding and supporting the access network part for V2X services.

##### RAN Aspects Support for the 4G & 5G V2X

4G access network is supported by the new radio (5G-NR) features for enhancing the ecosystem of V2X services. The 3GPP technical document (*TR 37.985 Rel-16*) reports overall RAN aspects: specifications, features design and operations, and interfaces (Uu and PC5 to cover communication types) for 4G & 5G V2X services and applications. To expand the support, the report also elaborates on resource allocation, congestion control, physical layer, higher-layer protocols, network aspects, and V2X via the Uu interface for 4G V2X and 5G V2X services implying that, each technology is considered for the given operations. Many use case scenarios are now considering both 4G and 5G radio-based communication networks working together and forming a multi-RAT (radio access technology) V2X. Hence, the existing scenarios consider both 4G & 5G V2X sidelink communications. This dictates the support for aV2X scenarios grouped into platooning, extended sensors, advanced driving, and remote driving as documented in the technical specification (*TS 22.186 Rel-16*). We would like to draw the attention of the reader from the automotive industry to these developments.

## 5. SA-Based 5G V2X Services

In the next generation of vehicular communication for the V2X services, the 3GPP has made significant contributions. In this regards, SA-based 5G communication technology is envisioned for future V2X communication in various technical reports and specifications of *Rel-16* and *Rel-17*. With SA-based 5G support for V2X services, the future V2X use case scenarios are possible. It goes without saying that the current support will be evolved in future releases i.e., *Rel-18* and *Rel-19* of 3GPP standardization. In what follows next, we analyze how SA-based 5G communication technology is enabling the V2X services and the success stories associated with them.

### 5.1. System Support

The next generation (5G) communication support is without a doubt the most valuable and significant support for V2X services offered by 3GPP. This introduced enhanced supports, which play important roles in realizing 5G V2X services i.e., non-safety (comfort), safety-related, and in multiple RATs (including non-3GPP V2X technology e.g., ITS-G5, DSRC, etc.). Obviously, for the evolved presentation of system support, the previously discussed service and system supports are considered for 5G V2X services. We now present a significant overview of 5G-based service and system support for V2X services.

#### 5.1.1. Enhancements Support

The technical report (*TR 22.886 Rel-15*) identifies various use cases and potential requirements for eV2X and aV2X services. The extended technical report (*TR 22.886 Rel-16*) complements the stated use cases and offers various new items targeting QoS due to the exposure of 5G communication technology. These updated changes are QoS change for remote driving applications; QoS list to support V2X critical applications; QoS estimation for different V2X applications; QoS aspect for extended sensors; corrections to use cases; and clarification on details of estimation information for different V2X applications. This implies that the V2X user experience of service is supported by QoS to better serve the needs of the V2X application. The V2X applications still need improved safety and non-safety services. Therefore, the latter version of (*TR 22.886*) analyzes use cases focusing on the interaction between the 3GPP system and the V2X application. In addition, another important use case is considering interoperability with non-3GPP V2X technologies.

The release 17 (*TR 21.917*) specifies NR PC5 V2X communication with respect to power efficiency for UE’s. It also adds support for QoS-aware NR-PC5 pedestrian UE power efficiency. It builds on the specification (*TS 23.287*) of PC5 Discontinuous Reception (DRX) operations. NR-based unicast, groupcast, or broadcast mode communications on PC5, i.e., PC5 DRX operations are now enabled with pedestrian UE power saving.

#### 5.1.2. Architectural Enhancements Support

The 5G-enabled concepts for V2X services are up-to-the-minute and require architectural enhancements in order to support V2X services. Apart from the architectural enhancements discussed in technical specifications (*TS 23.285*) and service requirements presented in technical specifications (*TS 22.185* and *TS 22.186*), 5G-specific architectural enhancements are required for 5GS to facilitate vehicular communications over 5G PC5 and Uu reference points (chosen independently when transmitting and receiving messages). In this regard, the technical specification (*TS 23.287 Rel-16*) provides architectural models and concepts i.e., reference model, functional entities, V2X application server, service parameters, reference points, and service-based interfaces. In addition, it specifies high-level functionality and features i.e., authorization and provisioning for V2X communications over PC5 and Uu reference points, V2X application server discovery, QoS handling for V2X communication, subscription to V2X services, and interworking between EPS-V2X and 5GS-V2X services. To accomplish the stated enhancements, various procedures for service authorization and provisioning to UE; V2X communication over PC5 and Uu reference points, and service authorization to NG-RAN for V2X communications over PC5 reference point are provided under functional descriptions and information flows of the specification. These enhancements and procedures motivate the automotive research community to contribute to the realization of the next-generation V2X services.

#### 5.1.3. Application Layer Support

The advancements promised for future generations are not only happening in communication technologies but in V2X scenarios focusing on real-world use cases. In Section 4, we discussed functional architecture, procedures, and information flows from technical document (*TS 23.286 Rel-16*). Considering the era of 5G, we present VAE layer support for 5G V2X services from the technical specification (*TS 23.286 Rel-17*). This specification considers existing studies i.e., *TR 23.795*, *TS 22.185*, and *TS 22.186*, etc., for architectural requirements, functional models, deployment models, identities, procedures and information flows, and VAE layer APIs. In addition, it provides the VAE capabilities application to EPS and 5GS.

The *Rel-17* document provides updates to the technical specifications such as V2X UE registration enhancement; V2X UE identity; session-oriented services; UE initiated session-oriented service; network monitoring procedure enhancement; UE-to-UE broadcast/groupcast configuration by VAE layer; support for enhancements to V2X group management and group communication; V2V communication mode switching; support for HD map dynamic information; dynamic local service information in multiple V2X service provider; V2X application layer architecture enhancement; PC5 Provisioning in multi-operator V2X scenarios; V2X service discovery across multiple V2X service providers; clarifications for network monitoring information notification; clarifications on network monitoring; business relationships between V2X service providers; monitoring and control of QoS for eV2X communications; and update to VAE server APIs.

Rel-17 (*TR 21.917*) specifies updates to technical specifications. These are; V2V communication assistance when mode switching to enable provisioning the V2X UE to use V2V communication modes switching from V2X application-specific layer. Cross-service provider discovery for V2X to allow V2C UE’s to find V2X services from a service provider while roaming outside the primary service provider coverage area. Retrieving dynamic local service information from a V2X UE through a service provider. Dynamic group information update for V2X platooning. The multi-agent scenario that supports PC5 provisioning for V2X/V2I communication. HD map dynamic information to enable V2X HD map server that obtains dynamic object information. V2X application server accesses the VAE layer services to disperse UE to UE groupcast/broadcast policy configurations and messages that are now enabled by UE to UE groupcast/ broadcast configuration and messages delivery services. V2X communications using local MBMS via the VAE layer are now supported. ToD application session management requirements are served using session-oriented services, the ToD may rest in UE or inside an application server. Extended QoS and Service adaptation is used for simplified service requirement adaptation service that is geared toward the V2X application server as the details of 3GPP system interactions are abstracted for the user.

### 5.2. Network Support

The present-day 3GPP releases are offering next-generation (5G) communication-based network support for addressing V2X services. In fact, the 5G design and architecture allow huge network efficiency and traffic capacity with the lowest latency in comparison to the 4G communication technology. Since, the 5G system includes a 5G core (5GC) and 5G access network (5G-AN), the 5G network support is divided into core network support and access network support. We now focus on the important parts of the core and access network support to equip the readers with the latest information regarding the support for V2X services.

#### 5.2.1. Core Network

The heart of the 5G network is 5G Core (5GC) used for establishing secure connectivity and access to services. Hence, 3GPP is using the new architecture (including network function (NF)) for core network support through reporting and specifying technical documents. Currently, these documents focus on the 5G system (5GS) and UE policies in 5GS. In what follows next, we provide an interesting discussion over the core network support.

##### 5G System Support

The 5G system plays an important role in specifying the V2X communication and configuration. The technical specification document (*TS 24.587 Rel-16*) discusses the protocol aspects for V2X services (in terms of messages transport) in 5GS and interworking to EPS considering the architectural enhancements of technical specification (*TS 23.287*). The transmission of V2X messages occurs over PC5 and/or Uu interfaces. In this connection, two protocols for data transmission and V2X communication are specified: among the UEs over the PC5 interface; between the UE and V2X application server over the Uu interface. Apart from the procedures described in the specification, it includes message functional definition and contents; information elements coding; coding other than information element coding; and a list of system parameters. In *Rel-17*, the updated changes involve security context identity for PC5 unicast, mutual authentication for PC5 unicast link, PC5 unicast link establishment for broadcast, alignments for providing an indication of activation of the PC5 unicast signaling security to lower layer, etc.

##### UE Policies in 5GS

The 5G-enabled V2X communication messages are transmitted and received to and from UEs following certain important and actionable policies as described in the technical specification (*TS 24.588 Rel-16*). For the UE policies configurations in 5GS, the stated specification considers the architectural enhancements support (discussed in *TS 23.287 Rel-16*) and the protocol aspects in 5GS (discussed in *TS 24.587 Rel-16 and Rel-17*). Furthermore, the latter documents also define the UE policy procedures for interfaces i.e., V2X communication over PC5 and Uu. For V2X communication over the given two interfaces, the encoding of UE policies is specified in the document (*TS 24.588*). Apart from the given specification, the UE policy delivery service is specified in (*TS 24.501*), which helps in providing the UE policies to the UE e.g., UE PC5 unicast signaling security policy, UE PC5 unicast user plane security policy, etc. The research community focusing on proposing the UE policies in 5GS is encouraged to follow the given descriptions and encoding schemes for V2X communication over PC5 and Uu interfaces.

#### 5.2.2. Access Network

The 3GPP standardization has considered 5G radio frequencies as enablers for 5G-V2X communication. In this connection, various standard documents are reported and specified providing 5G-enabled access network support for V2X services. Including other network supports, two important RAN supports are reported for NR support and UE radio transmission and reception.

##### New Radio Support

The 5G new radio (5G-NR) is expected to provide support for the current aV2X use cases (as detailed in (*TR 22.186 Rel-15 and Rel-16*)) and new aV2X services. In this regard, the 3GPP technical report (*TR 38.885 Rel-16*) investigates the 5G RAN aspects for advanced V2X services, including 5G sidelink (PC5) aspects, i.e., NR sidelink design, synchronization, resource allocation, and protocols; Uplink and downlink (Uu) aspects, i.e., aV2X use cases over Uu interfaces, and resource allocation/configuration; QoS management, i.e., model, profile, and parameters; RAT and interface selection, i.e., 4G and/or 5G RAT, UE upper layers select the radio interface; coexistence, i.e., between 4G and 5G V2X; network aspects, i.e., authorization, slicing aspects, and resource coordination; and evaluations and measurement results.

##### UE Radio Transmission and Reception Support

The core and access network part of 5GS has been contributing to the realization of advanced V2X use cases available in *Rel-15* and *Rel-16*. Though the UE policies in 5GS cover the core network part, the UE radio transmission and reception for 5G-based V2X services are reported in the technical document (*TR 38.886 Rel-16*) for covering the 5G access network part. The reported radio solutions provide further 5G network support to the described aV2X services in the document (*TR 38.885 Rel-16*) [21]. Hence, the 3GPP reported supports for V2X service are: 5G sidelink operations and QoS management; operating bands and channel arrangements; transmitter and receiver characteristics; and evaluation of channel and in-device coexistence. The available specifications and recommendations will help NR V2X operations and allow new radio frequency-based solutions from the relevant research community. The researchers are also encouraged to make use of radio frequency core requirements for 5G V2X operating scenarios and plan futuristic procedures for realizing 5G V2X services.

## 6. Network Data Analytics Function (NWDAF)

The Network Data Analytics Function (NWDAF) was introduced in 3GPP *Rel-15* [22] as a new network function of the 5G core architecture. It was subsequently enhanced and detailed in 3GPP *Rel-16* and *Rel-17*, including some early use cases. Although the idea to ingest analytics to the mobile core network is not new, it lacked standardization that would have enabled better adoption. It was previously done through a custom approach driven by specific vendor implementations. The 3GPP NWDAF is an attempt to standardize the way to do analytics in the 5G core network as well as leverage machine learning operations (MLOps) best practices. The NWDAF can interface with various control and data plane network functions to collect events that are of interest for analysis to monitor, for example, compliance with relevant service-level agreements (SLAs), or to intelligently optimize the network resources. The NWDAF includes one or more of the following functionalities:

- Support data collection from network functions (NFs) and analytical functions (AFs),
- Support analytics information provisioning to NFs and AFs,
- Support machine learning (ML) model training and provisioning to NWDAFs (containing analytics logical function).

The architecture of NWDAF is defined in the technical document (*TS 23.288*) [23] and the detailed specification with APIs, etc., is defined in the technical document (*TS 29.520*) [24], whereas the NWDAF use case is defined in the technical document (*TS 23.791*) [25]. The NWDAF is expected to have a distributed architecture providing analytics at the edge in real-time and a central function for analytics that need central aggregation (e.g., service experience). Therefore, the implementation of NWDAF should happen in the SA-based 5G with its service-based architecture. The NWDAF collects data and provides analytics services using a request or subscription model. It means, any consumer NF, such as PCF, AMF, or NSSF, should first subscribe to the analytics service with agreed data inputs from the consumer and return analytics or ML model inference results. The types of observed events by NWDAF include:

- Slice load level information,
- Network slice instance load level information,
- Service experience,
- Network Function (NF) load,
- Network performance,
- Abnormal behavior,
- UE mobility and UE communication,
- User data congestion, and
- QoS sustainability.

The above events give an idea of how NWDAF can be powerful to advance the network automation that is needed for complex use cases such as autonomous driving. An accurate prediction of the network slice can be translated into a proactive adjustment for the network slice and optimized resource allocation.

### 6.1. NWDAF and Federated Learning

Given the distributed nature of NWDAF implementation, a new challenge occurs, which is the data privacy being transmitted as events. Moreover, the volume of this data is huge, and transferring the data as is will cause a dent in the latency. Therefore, we are observing a trend in research by combining the NWDAF implementation with federated learning (FL).

**Figure 4:**
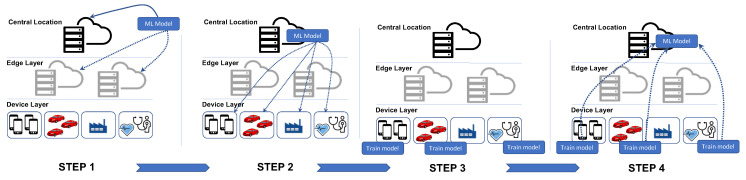
Steps of Federated Learning.

The FL has the potential to address privacy challenges and communication congestion [26]. It allows stakeholders, in this case, the NFs, to train the model with their local datasets within their own premises without the need to share the raw data with any other entity. The generated hyperparameters/gradients of their locally trained model get sent to the orchestrating/central server [27], which is responsible for aggregating these parameters from different stakeholders. The server keeps updating the central model by capturing hyperparameter values from the clients. This is to say that each client feeds into the global model at the central server and then downloads the updated global model. The central server keeps iterating this process until the learning is matured [28]. Mobile Edge Computing (MEC), on the other hand, has further added value to the impact of FL by enabling the placement of the central model nearer to the data source layer, which is an important achievement when it comes to realizing more complex use-case scenarios requiring active learning and real-time decisions for critical maneuvers. Please refer to Figure 4 for high-level steps of FL.

Few researchers started to implement the NWDAF with or without the leverage of FL. The authors in [29] provide an implementation result of NWDAF in free5GC, i.e., open software for 3GPP mobile CNs. Their implemented NWDAF module consists of: (1) model training logical function (MTLF) to train the model; and (2) analytics logic function (AnLF) to provide analytic results based on the trained model. The code is available on GitHub. While the authors in [30] introduces a distributed NWDAF structure tailored for FL in 5G. Distributed NWDAFs are largely composed of a single root NWDAF and several leaf NWDAFs, and an interface exists among distributed NWDAFs to enable communication with each other. It is assumed that the root NWDAF is installed at the central cloud and each leaf NWDAF is installed at the edge cloud in a container form for each NF. However, the paper only presents a design for the use case with no backed experiment.

**Figure 5:**
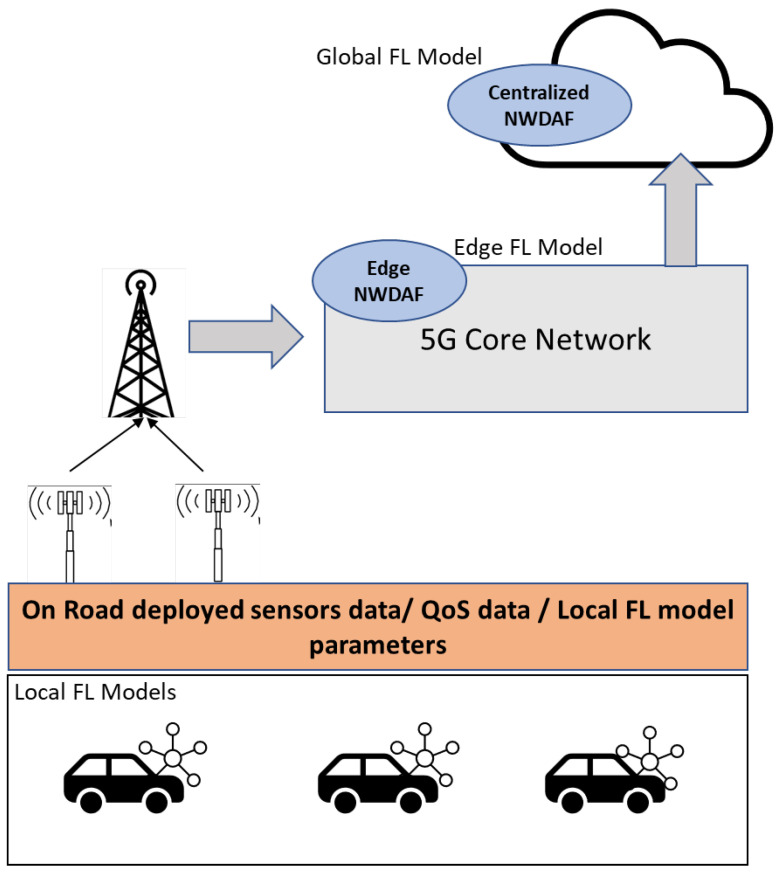
High-level design of our NWDAF deployment.

On the other hand, the authors of [31] have designed a closed-loop Intent-based Networking (IBN) platform leveraging NWDAF. They developed and tested two models, one to detect and prevent anomalies, and another to predict the network slice load. The decision to reallocate the resources by the IBN platform is fed by the inference of these models. Another usage of NWDAF to detect the load of the future network slice found in [32]. The authors used a centralized NWDAF and common ML models to detect the load. This research work [33], however, leverages NWDAF for another use case, namely, determining the appropriate value of inactivity timers associated with each PDU session in 5G networks. They used a centralized NWDAF with plain data. The authors propose a deep reinforcement learning (DRL) approach to optimize the inactivity timer value on each 5G PDU session of UEs. The recent introduction of NWDAF and the 3GPP to standardize data analytics in 5G+ networks has inspired us to focus on building an intelligent service orchestrator to achieve a higher level of autonomy in the network. Our solution leverages NWDAF, Edge Computing, and FL. Figure 5 depicts the high-level design of the NWDAF deployment that supports the service orchestrator. The concept is based on merging both the vehicle-applications data with the QoS data and road data together to support the network with the right decision on the service.

### 6.2. Challenges of Federated Learning

Although FL addresses the crucial requirement of preserving privacy, a number of challenges still have to be addressed for FL to achieve its full potential. The following is a list of major challenges. For a detailed literature review of the work done addressing these challenges, we refer to our paper [34]

#### 6.2.1. Heterogeneous Characteristics of Clients

The central servers in the classical ML approaches are equipped with rich computation and storage infrastructure. Clients may have differing characteristics (e.g., storage, computation power, and communication capabilities including interfaces, throughput, battery life, etc.), of edge and device layer entities including smartphones, vehicles, body area sensors, industrial sensors, etc. This becomes a challenge in FL, as this may result in varying learning times and resource availability. Application domains with many clients usually suffer from such heterogeneously equipped device layers, resulting in asynchronous updates toward the server. This introduces delays in aggregation at the server.

#### 6.2.2. Achieving Reliable and Dynamically Configurable Communication Bit-Pipes

FL involves three major steps: Step 1—clients download the global model; Step 2—clients train the model with their local datasets; and Step 3—clients upload a global model with their locally trained models. In the classical FL, all the clients need to communicate their full gradient (e.g., in GBs) update for each epoch. These steps are looped until the convergence of a global model is achieved. Such inter-client server interaction asks for QoS-guaranteed communication bit-pipes. The issue gets massive in situations where the clients are highly mobile, e.g., autonomous driving, mobile networks, etc. To capture the communication link requirements, we consider the utility function U(.) that captures the type of service, type of user, and communication-specific parameters. This function is adapted from the author-proposed user satisfaction function in [35]. Let $u_{j,k,c}\left(n\right)$ represents the satisfaction function of user *j*, the proposed utility function takes care of service relevant requirements, and is given by:

(1) $$\begin{array}{c} u_{j,k,c}\left(n\right):={\bar{u}}_{j}\left(\frac{b_{k}^{c}}{n_{k}}\right)\prod_{l\in L}{\left(\nu_{jl}\left(k,c,n\right)\right)}^{w_{jl}}\\ +\sum_{l^{\prime }\in L^{\prime }}\omega_{l^{\prime }}v_{jl^{\prime }}\left(k,c,n\right), \end{array}$$

We decompose the user utility function into four components:

- The term ${\bar{u}}_{j}\left(\frac{b_{k}^{c}}{n_{k}}\right)$ is the function of network state $n={\left(n_{k}^{c}\right)}_{k,c}$ and the offered bandwidth $b_{k}^{c}$. The collection *n* is the vector that represents the total number of users who request the service of a specific class.
- $\prod_{l\in L}{\left(\nu_{jl}\left(k,c,n\right)\right)}^{w_{jl}}$ is the weighted multiplicative approach for *bandwidth-dependent* associated QoE attributes, i.e. delay, packet loss, etc.
- $\sum_{l^{\prime }\in L^{\prime }}\omega_{l^{\prime }}v_{jl^{\prime }}\left(k,c,n\right)$ is the weighted sum of different *independent* QoE attributes.

For more details on the user satisfaction function, readers are encouraged to refer to [36,37]. We further assume that the process of handover incurs costs, which we capture in a similar fashion as proposed in our earlier work. More on this may be found in our work in [38]. As mobile users’ channel conditions vary, the service offers of network technologies (i.e., offered bandwidth with associated QoS parameters) need to meet the users’ required service parameters. We represent such parameters by a utility function $u_{j}$ for host *j* as proposed in our earlier work [36]. The proposed utility function is a quasi-concave function that realistically captures user satisfaction on offered bandwidth, QoS indices (e.g., delay, jitter, packet loss) of offered network service, and service costs.

#### 6.2.3. Varying Client Sets

The assumption that all the clients remain active all the time may not hold in many cases. For instance, in autonomous driving settings, the clients, i.e., autonomous vehicles, in specific regions may not be part of FL for every iteration. Hence, a more realistic assumption is that only a fraction of the clients will be part of the learning at a particular time. Furthermore, those active clients may also drop out of the learning process due to poor connectivity or inadequate computing resources. Major problems under this category are accurately estimating active clients and creating a framework supporting hardware with heterogeneous characteristics.

#### 6.2.4. Statistical Heterogeneity

With varying data from the involved clients, the distribution is usually non-identical. This is because the clients capture or generate data in a non-identically distributed fashion across the domain. For example, in eHealth settings, the data captured via wearable devices vary from individual to individual. In autonomous driving applications, the vehicles that are from different automobile makers and differently digitized will capture in a non-identically distributed manner. Furthermore, the number of data points for different devices may significantly vary, which negates the independent and identically distributed (I.I.D.) assumptions usually used in distributed optimization. It also increases the probability of stragglers and complexity.

#### 6.2.5. Privacy Concerns and Data Labeling

For privacy concerns, the FL does move in the direction of ensuring the privacy of data by sharing the hyperparameters or gradient information rather than the raw data. However, this may reveal sensitive information to the central server or other stakeholders by using model inversion techniques. On the other hand, data labeling and pre-processing are important stages of machine learning. For instance, the supervised learning models demand that data is clearly labeled. This is obviously challenging to achieve across various clients of FL. Hence, it is imperative to design and develop model data pipelines that apply labels in a standardized way, based on events and user actions.

#### 6.2.6. Model Convergence Time

The convergence time of FL is typically longer than that of the locally trained models. The factor of unpredictability fueled by the challenges mentioned above includes unreliable connection, heterogeneous devices, varying software versions, varying applications, etc.; these all add to the complexity and, consequently, to the convergence time. For this reason, FL solutions are typically most useful when they provide meaningful advantages over centrally training a model, such as in instances where datasets are extremely large and distributed.

#### 6.2.7. Personalization

The QoE is highly associated with how a service accommodates the unique needs of the user. Personalization is the key to a better user experience. In FL, where all users will receive the same global model, some personalization can be lost in the middle. Therefore, finding solutions to address the personalization matter is important.

#### 6.2.8. Incentivization

In FL, model convergence takes longer when compared to centralized training models. The potential for large companies to centrally aggregate data and create silos for competitive advantage could be an obstacle to FL adoption. Besides the time factor, there is a quality factor that can demotivate nodes to choose FL. Specifically, when local datasets are small and the data distribution is I.I.D, global models typically outperform local models, and the majority of clients benefit from participating in the FL process. However, when clients have significantly large private datasets and the data distribution is non-I.I.D., local models exhibit better performance than that of the shared global model, and clients have no incentive to participate in the FL process. Effective data protection policies and appropriate incentives around data cooperation can tackle these issues and develop a beneficial ecosystem. Considerable effort is needed to create FL systems that protect intellectual property and incentivize widespread use and adoption.

## 7. Future Directions

The challenges of FL asks for new solution approaches in the near future. Since AI and ML are newly introduced in the 3GPP’s releases and technical documents, it would become interesting to find a joint solution approach that introduces intelligence in vehicles. Therefore, matching intelligence would be required in the network and on the road when compared to the ever-increasing intelligence developed by automotive OEMs inside the vehicles. Whether it is autonomous driving, eHealth, or industry 4.0 applications, CSPs must advance the automation level of their networks to cope with the stringent requirements of these applications such as ultra-low-latency and dynamic provisioning for agreed SLAs. The recent introduction of NWDAF and the pursuit of researchers and CSPs to standardize data analytics in 5G+ networks has inspired us to focus our research work on building an intelligent service orchestrator to achieve a higher level of autonomy in the network. Our solution leverages NWDAF, Edge Computing, and FL. We presented the high-level design of the NWDAF deployment that supports the service orchestrator. The concept is based on merging both the vehicle-applications data with the QoS data and road data together to support the network with the right decision on the service. The local FL models reside in the vehicle. It can be a model that predicts the next maneuver or next destination. These models will send the parameters to the edge NWDAF, along with other needful data from the road and also the QoS data. The edge NWDAF will host multiple edge FL models, each for a different purpose, and serving that particular area of the road. For example, something like adjusting the service as per SLA, or notification for the vehicle with the better route alternative to take based on other data coming from the road. The centralized NWDAF, on the other hand, resides in the cloud and hosts global FL models. Each of these global models has a specific purpose and is the result of federating the edge FL models from the edge NWDAF. For example, one global FL model can be responsible for predicting the network slice load. The prediction will help the network to proactively re-configure the slice to remain serving as per the SLA.

## 8. Conclusions

The C-V2X applications are gaining attention from both academia and industry to realize Level-5 autonomous driving. In this context, the 3GPP has been supporting V2X services to realize advanced V2X applications. This paper provides an exhaustive analysis of the 3GPP C-V2X evolution from 4G V2X to the state-of-the-art 5G V2X as from *Rel-14* to *Rel-17* elaborating on the 3GPP standardization support for the system, core, and radio network. We focused on providing a complete support package for the research community to start considering the 5G mobile technology for a higher level of V2X applications. To guide the readers over the 3GPP support for V2X services, we discussed the communication requirements, use cases, key issues, and the potential solution for each communication technology i.e., 4G, NSA-based 5G, and SA-based 5G. Furthermore, we discussed the architecture, implementation, and challenges of NWDAF along FL. This was geared towards helping the researchers to have a holistic view of the current trends and easily focus on addressing interesting yet important issues and challenges. We believe, the paper will significantly assist the researchers in quickly identifying relevant documents of interest from the standards and better understanding the technical specifications aiming to deploy C-V2X services and applications.

## Figures and Tables

**Table 1:** 3GPP Standardization Support for C-V2X Services.

Technology Categorization	Document ID	Topic	Release-14		Release-15		Release-16		Release-17	
			Version	Year	Version	Year	Version	Year	Version	Year
**4G V2X** **Services**	TS 22.185	Requirements for V2X Services	14.4.0	2018-06	15.0.0	2018-06	16.0.0	2020-07		
	TR 22.885	LTE support for V2X Services	14.0.0	2015-12						
	TS 23.285	Architecture enhancements for V2X Services	14.9.0	2019-12	15.4.0	2019-12	16.4.0	2020-09		
	TR 23.785	Architecture enhancements for V2X Services	14.0.0	2016-09						
	TR 23.795	Application Layer for V2X Services					16.1.0	2018-12		
	TS 24.385	Management Object (MO) for V2X Services	14.4.0	2018-09	15.1.0	2018-09	16.2.0	2020-09		
	TS 24.386	Protocol Aspects for UE to V2X Control Function	14.5.0	2020-06	15.3.0	2020-06	16.2.0	2020-12		
	TS 24.486	Protocol Aspects for V2X Application Enabler (VAE) Layer					16.3.0	2021-03		
	TS 29.387	V2X Control Function to V2X AS Aspects	0.1.0	2016-11						
	TS 29.388	V2X Control Function to HSS Aspects	14.2.0	2019-09	15.1.0	2019-09	16.0.0	2020-06		
	TS 29.389	Inter-V2X Control Function Signalling Aspects	14.2.0	2019-09	15.1.0	2019-09	16.0.0	2020-06		
	TR 33.885	Security Aspects V2X Services	14.1.0	2017-09						
	TR 36.786	UE Radio Transmission and Reception for V2X	14.0.0	2017-03						
	TR 36.787	New Band Combinations for V2X			15.0.0	2018-07				
	TR 36.788	UE Radio Transmission and Reception for V2X			15.0.0	2018-07				
	TR 36.885	LTE-based V2X Services	14.0.0	2016-07						
**4G** & **NSA-based 5G** **V2X Services**	TR 22.886	Enhancement of V2X Services for 5G			15.3.0	2018-09	16.2.0	2018-12		
	TS 23.286	Functional Architecture and Information Flows for V2X Services					16.5.0	2020-12	17.1.0	2021-04
	TR 23.764	Enhancements to Application Layer for V2X Services							17.1.0	2020-12
	TR 23.776	Architecture Enhancements for aV2X Services							17.0.0	2021-03
	TR 23.786	Architecture Enhancements of aV2X Services for the EPS and 5GS					16.1.0	2019-06		
	TR 26.985	Media Handling and Interaction for V2X			0.2.1	2018-01	16.0.0	2019-12		
	TS 29.486	V2X Application Enabler (VAE) Services					16.3.0	2021-03	17.0.0	2021-03
	TS 33.185	Security Aspect for V2X Services	14.1.0	2017-09	15.0.0	2018-06	16.0.0	2020-07		
	TS 33.536	Security Aspects aV2X Services					16.3.0	2021-03		
	TS 33.836	Security Aspects aV2X Services					16.1.0	2020-09		
	TR 37.875	Band Combinations for Uu and V2X con-current Operation							0.4.0	2021-06
	TR 37.985	RAN Aspects for V2X based on LTE and NR					16.0.0	2020-07		
**SA-based 5G** **V2X Services**	TS 23.287	Architecture Enhancements of V2X Services for 5GS					16.5.0	2020-12		
	TS 24.587	V2X Services in 5GS					16.4.0	2021-03	17.1.0	2021-03
	TS 24.588	UE Policies for V2X Services in 5GS					16.4.0	2021-03		
	TR 38.885	NR-based V2X					16.0.0	2019-03		
	TR 38.886	UE Radio Transmission and Reception for NR-based V2X					16.3.0	2021-04		
**V2X Scenarios and Use Cases**	TS 22.186	Enhancement for V2X Scenarios			15.4.0	2018-09	16.2.0	2019-06		
	TR 37.885	Evaluation Methodology of new V2X Use Cases for LTE and NR			15.3.0	2019-06				

**Table 2:** General and Scenario-based Performance Requirements to Support Advanced V2X Scenarios.

5G Use Cases				Performance Requirements					
			**Degree of ** **Automation**	**Payload** **(Bytes)**	**Tx Rate** **(message/** **sec)**	**E2E** **Latency** **(ms)**	**Reliability**	**Data** **Rate** **(Mbps)**	**Comm. ** **Range** **(m)**
		**General Requirements**		50–6000	30	10–500	90–99.99	50–65	80–350
**Platooning**	**Scenario** **Specific**	Vehicular Platoon drives cooperatively and exchanges information between groups of UEs supporting V2X services.	Lowest	300–400	30	25	90	-	-
			Low	6500	50	20	-	-	350
			High	-	-	20	-	65	180
			Highest	50–1200	30	10	99.99	-	80
		Reporting needed for platooning between UEs supporting V2X application and between a UE supporting V2X application and RSU.	-	50–1200	2	500	-	-	-
		To share information for platooning between a UE supporting V2X application and RSU.	Lower	600	50	20	-	-	350
			Higher	-	-	20	-	50	180
		**General Requirements**		1600	-	3–100	90–99.999	10–1000	50–1000
**Extended** ** Sensors**	**Scenario** ** Specific**	Sensor information sharing between UEs supporting V2X application	Lower	1600	10	100	99	-	1000
			Higher	-	-	10	99.99	25	500
		Video sharing between UEs supporting V2X application	Lower	-	-	50	90	10	100
			Higher	-	-	10	99.99	90	400
		**General Requirements**		-	-	5	99.99	UL: 25 DL: 1	-
**Remote ** ** Driving**	**Scenario** ** Specific**	To exchnage information between a UE supporting V2X application and a V2X Application Server	-	-		5	99.99	UL: 25 DL: 1	-
**Advanced ** ** Driving**	**General Requirements**			2000–12,000	100	3–10	99.99	30–53	500

## Data Availability

Not applicable.

## Abbreviations

The following abbreviations are used in this manuscript:

AD	Autonomous Driving
AV	Autonomous Vehicles
3GPP	3rd Generation Partnership Project
OEM	Original Equipment Manufacturers
4G	4th-generation
5G	5th-generation
V2X	Vehicle to Everything
C-V2X	Cellular-V2X
LTE-V2X	long-term-evolution-V2X
V2V	Vehicle to Vehicle
V2P	Vehicle to Pedestrian
V2N	Vehicle to Network
V2I	Vehicle to Infrastructure
ITS	Intelligent Transportation Systems
CAV	Connected and Automated Vehicles
DSRC	Dedicated Short Range Communication
Rel-14	Release 14
eV2X	Enhanced-V2X
aV2X	advanced V2X
5GS	5G System
5GC	5G Core
NR	New Radio
TR	Technical Report
TS	Technical Specification
NWDAF	Network Data Analytics Function
NF	Network Function
FL	Federated Learning
MLOps	Machine Learning Operations
